# Association between physical activity and taste–The advantage of increased intensity for some but not all individuals

**DOI:** 10.1371/journal.pone.0295173

**Published:** 2023-12-27

**Authors:** Alexandre-Charles Gauthier, François Dupont, Marie-Eve Mathieu

**Affiliations:** 1 School of Kinesiology and Physical Activity Sciences, Faculty of Medicine, Université de Montréal. Montréal, QC, Canada; 2 Sainte-Justine University Hospital Center, Montréal, QC, Canada; University of Montenegro, MONTENEGRO

## Abstract

**Introduction:**

Taste is a key sensory modulator of energy intake, and while the effects of acute physical activity (PA) on taste have been recently confirmed, its chronic effects remain poorly documented.

**Methods:**

Data were extracted from the NHANES database, including salty and bitter taste tests, moderate PA (MPA) and vigorous PA (VPA) levels, and anthropometric data. Binary logistic regressions were conducted, and odds ratios (ORs) represent the association between physical activity level and successful taste tests.

**Results:**

3,114 participants (51.5% women, mean age 58.5±11.9 years, mean BMI 29.5±7.0 kg/m^2^) were analyzed. For the tongue tip test, the frequency of VPA was associated with a better score for the bitter and total taste test, while duration of VPA was associated with a better score for the bitter, salty, and total taste test (OR [1.01–1.06], p<0.05). For the whole mouth test, MPA frequency improved the bitter taste score (OR = 1.06, p = 0.01) while VPA frequency and duration were associated with better scores for bitter, salty, and total taste tests (OR [1.01–1.15], p<0.05). These findings were distinctively influenced by weight status and gender, with males and individuals without obesity mainly beneficiating from an active lifestyle.

**Perspectives:**

This study underlines the link between PA, particularly its frequency and intensity, and improved taste preservation. These findings emphasize the potential benefits of regular VPA for optimizing taste perception, although questions remain regarding the advantages for individuals with obesity and the lack of association observed in women who might already have a preserved profile.

## 1. Introduction

Chemosensation refers to the chemical response to a stimulus [1]. When this response is reduced, it can lead to an increase in the detection threshold for smell and taste. Such a condition is not without consequences since taste plays a crucial role in nutrient sensing, food consumption, and satiety [2, 3]. Not surprisingly, people with altered taste perceptions often face inadequate food consumption including malnutrition and are more prone to food-related [4]. This is often the case for the elderly in which a diminution in chemosensory function is found [5].

In the past few years, body weight status, especially obesity, has been linked to altered taste perceptions [6, 7]. In fact, decreased taste sensitivity has been observed in people with higher body mass indexes (BMI) when compared to a population with normal body weight [8, 9]. Differences in taste perceptions have also been observed when comparing male and female participants who had their taste sensitivity assessed for sweet, salty, sour, and bitter tastes [10]. In general, women exhibit a better taste recognition threshold for salty, sour, and bitter tastes [10]. Recently, Rosa and colleagues reported the same pattern; better taste intensity ratings observed in female participants when compared to male participants [11]. Interestingly, they observed that higher body weight in women was also negatively associated with the intensity ratings of both salty and bitter taste, with better results obtained in the group weighing under 60kg compared to the group weighing over 60kg [11].

A single session of physical activity (PA) has a documented impact on taste perceptions. According to a recent systematic review conducted by our group, acute PA affects taste detection and perception differently depending on the type of taste [12]. For example, acute PA seems to enhance sweet taste preference, sensitivity, and intensity. In return, chronic PA lowered preference for sweet foods. As for salty taste, taste intensity perception is lowered during acute PA and seems to be impacted by its duration, with longer PA periods associated with a greater impact on taste intensity. Salty taste sensitivity is also reduced following acute PA, while preference is increased [12]. As for the relationship of acute PA with other tastes such as bitterness, umami, and sourness, the literature remains limited. In our review, 16 out of 18 studies presented results for sweet taste, 10 out of 18 for salty taste, and only 5 out of 18 for bitterness, umami, and sourness [12].

PA performed on a regular basis (i.e., chronic) could enhance senses and thus offers a safe and effective countermeasure to certain sensory losses [13]. Although acute PA has a documented impact on the gustative system, a relatively small body of evidence is currently available on the effect of chronic PA on taste. In a study on frequency of PA where active people, defined as ≥4 days/week of PA, were compared to a control group, defined as <1 day/week of PA, Feeney and colleagues reported a better identifying capacity for the exercising group for umami taste, higher perceived intensity ratings for sweet and umami tastes, and lower preference ratings for sweet and umami taste compared to the control group [14]. When looking at the other chemosensory sense (i.e., olfactory) in the National Health and Nutrition Survey (NHANES), moderate-to-vigorous PA practice was associated with lower olfactory dysfunction when practiced ≥3 days per week [15]. Therefore, it is legitimate to wonder if an active lifestyle is associated with reduced taste impairment in NHANES to enhance the body of literature available on the topic but also to go beyond the study of a singular threshold to assess the various components of PA. In addition, the various components of an active lifestyle (intensity, duration and frequency of PA) have never been directly assessed for its impact on taste.

Using NHANES, this study aims to determine if overall PA practice is associated with a better taste profile and if different components of chronic PA (intensity, frequency, duration) are associated with better taste perceptions. It is hypothesized that physically active people will have better-preserved taste perceptions compared to those who are not, and that PA frequency and intensity will be key determinants of PA’s effect. This study also seeks to explore whether there are notable differences in bodyweight status and sex regarding the chronic effect of PA on taste perceptions.

## 2. Methods

### 2.1 Study design

Data were extracted from the NHANES 2013–2014. NHANES is a program of studies designed to assess the health and nutritional status of adults and children in the United States. In 2013–2014, the impact of different PA levels on taste perceptions was assessed.

### 2.2 Participants

In the 2013–2014 NHANES cohort, participants had to be 40 years or older to be eligible for the chemosensory tests conducted by the examiners [16]. Exclusion criteria for the chemosensory tests were participants who were either pregnant or breastfeeding, and failure to adequately complete the test regarding LED light intensity (3 lights were presented to each participant, and the participants had to correctly rate the order of the LED intensity) resulted in exclusion from the dataset. These 3 light ratings were used to identify participants who were and were not able to use the scale gLMS accurately, which is the scale used in the taste tests. It is worth mentioning that participants who were allergic to quinine were excluded from the quinine taste test but completed the rest of the procedure [17]. To be included in the present study, complete data for the chemosensory tests, PA questionnaires and anthropometric results were required.

### 2.3 Anthropometric data

For each participant, different body measurements were obtained by the examiner team, which included body weight in kg, standing height in cm, waist circumference in cm, and body mass index (BMI) in kg/m^2^ [17]. For the current study, participants’ BMI was used as the main stratifying variable and was separated into four distinct categories: normal weight (≤24.99 kg/m^2^), overweight (25.00–29.99 kg/m^2^), obesity class 1 (30.00–34.99 kg/m^2^), and obesity class 2 and 3 (≥35.00 kg/m^2^).

### 2.4 Physical activity assessment

At-home self-reported PA questionnaires were used to assess participants’ overall PA levels using the computer-assisted Personal Interview system [18]. The questionnaire was based on the PA Global Questionnaire, which is derived from the Global Physical Activity Questionnaire, and included questions related to daily activities, leisure activities, and sedentary activities. To assess whether participants were physically active or not, they were first asked if they engaged in ≥10 minutes continuously each week, for both MPA and VPA individually. Then, to analyze the participants’ total MPA and VPA duration, blocks of 10 minutes per week were used to describe their weekly PA practice. MPA and VPA frequency were described as the number of active days per week, for ≥10 minutes continuously. PA intensity was set at 4 METs for moderate PA (MPA) and 8 METs for vigorous PA (VPA), as recommended in the NHANES PA Global Questionnaire [18].

### 2.5 Taste assessment

According to the NHANES official protocol, "The taste and smell exam measured the ability to taste and smell, using an odor identification test and salt and quinine taste testing" [16]. The taste test was separated into two distinct phases: Tongue Tip Taste Testing and Whole Mouth Taste Testing. The taste tests were conducted using the generalized Labeled Magnitude Scale (gLMS), an evaluation tool/questionnaire used to rate taste intensities ranging from "barely" to "strongest of any kind" [19].

In the first phase, participants were exposed to "Tongue Tip Taste Testing," which was conducted with two different tastants: 1 mM quinine (bitter) and 1 M NaCl (salty). They were asked to stick out their tongues while the researcher applied a cotton swab applicator covered with a tastant to the tip of their tongue. Subsequently, participants had to rate the intensity using the gLMS and identify the tastant that was presented to them. Participants were asked to keep their tongues out while assessing each tastant. Their mouth was thoroughly rinsed with water for 30 seconds in between the two tastants [17].

In the second phase, the "Whole Mouth Taste Testing" was conducted with three tastants: 0.32 M NaCl (salty), 1 mM quinine (bitter), and 1 M NaCl (salty). The tastants were presented in randomized order for each participant. Participants were asked to take a 10ml tastant solution in their mouth, swish the solution for 3 full seconds, spit it out, rate the intensity, and then identify the tastant that was presented to them. Their mouth was thoroughly rinsed with water for 30 seconds in between each tastant [17].

### 2.6 Statistical analyses

Age, BMI, and self-reported PA levels in terms of frequency and overall volume are presented as mean ± standard deviation. The impact of PA on taste test results was analyzed using chi-squared tests and binary logistic regressions. The odds ratio from the regression represented the effect of PA on the likelihood of success in the Tongue Tip Taste Test, Whole Mouth Taste Test, and overall success in both tests. Age and sex were controlled for in the analysis. Sub-analyses were also performed to identify differences regarding BMI and sex. Statistical analyses were performed using IBM SPSS Statistics 28^TM^ (Endicott, United States of America).

## 3. Results

### 3.1 Participants characteristics

Following the inclusion criteria for taste, the current sample includes 3,114 participants (51.5% female). The average age is 58.5±11.9 years with an average BMI of 29.5±7.0 kg/m^2^. On average, participants did MPA for 1.6±2.8 days per week for a total of 87.9±176.4 minutes per week and VPA for 0.60±2.28 days per week for a total of 38.4±124.5 minutes per week.

### 3.2 Taste results

#### 3.2.1 Tongue tip test

First, it was wondered if the practice of PA alone could be associated with preserved taste perceptions as measured by the tongue tip test. No significant results were obtained for the tongue tip test for MPA practiced for at least 10 minutes continuously/week vs no MPA practice (see Table 1). People who practiced VPA for at least 10 minutes continuously/week had significantly higher rates of success in all the tongue tip tests when compared to those who did not practice any VPA (all p values = 0.004).

**Table 1:** Moderate and vigorous physical activity practice and their association with the tongue tip test.

		Tongue tip bitter			Tongue tip salty			Tongue tip total		
		n	Success %	P value	n	Success %	P value	n	Success %	P value
**Moderate PA**	**Yes**	1343	40.4	0.281	1334	84.0	0.299	1334	35.6	0.490
	**No**	1771	38.4		1761	82.6		1761	34.4	
**Vigorous PA**	**Yes**	511	**45.0**	**0.004**	507	**87.6**	**0.004**	507	**40.4**	**0.004**
	**No**	2603	**38.1**		2588	**82.4**		2588	**33.8**	

PA: Physical activity

When investigating each PA intensity into its two features of interest (frequency and duration), no significant results were obtained with MPA for the tongue tip test as a whole (see Table 2). For VPA practice, frequency was associated with better results in both bitter (OR = 1.061, p = 0.020) and total score test (OR = 1.055, p = 0.035). Duration of VPA was associated with better results in bitter (OR = 1.007, p = 0.025), salty (OR = 1.013, p = 0.018) and total score test (OR = 1.006, p = 0.043) (see Table 2).

**Table 2:** Frequency and duration of moderate and vigorous physical activity for the tongue tip test.

		Tongue tip bitter			Tongue tip salty			Tongue tip total		
		n	OR [CI]	P value	n	OR [CI]	P value	n	OR [CI]	P value
**Moderate PA**	**Frequency (days/week)**	3114	1.017 [0.990–1.044]	0.217	3095	1.023 [0.981–1.067]	0.287	3095	1.016 [0.989–1.043]	0.244
	**Duration (10min blocs/week)**	3114	1.000 [0.996–1.005]	0.859	3094	1.001 [0.996–1.007]	0.703	3094	0.999 [0.995–1.004]	0.722
**Vigorous PA**	**Frequency (days/week)**	3114	**1.061 [1.009–1.115]**	**0.020**	3095	1.075 [0.999–1.158]	0.054	3095	**1.055 [1.004–1.109]**	**0.035**
	**Duration (10min blocs/week)**	3113	**1.007 [1.001–1.013]**	**0.025**	3094	**1.013 [1.002–1.023]**	**0.018**	3094	**1.006 [1.000–1.012]**	**0.043 **

PA: Physical activity

When stratified by BMI subgroups, participants who did MPA did not obtain better results for the bitter, salty and total score test (data not shown). In participants who did VPA, one significantly result was noted in the normal weight category for the bitter tongue tip test (OR = 1.100, p = 0.026).

When looking at the differences between men and women, both frequency and duration of MPA and VPA did not yield any significant results in the tongue tip test for the group comprised of only women (data not shown). For men, no significant results were obtained for both the frequency and duration for MPA practice while a significantly better taste capacity was noted with increased VPA duration for the salty taste with an OR = 1.017 (p = 0.016).

#### 3.2.2 Whole mouth test

Higher rates of success for the whole mouth bitter test (p = 0.046) and the whole mouth total score (p = 0.018) were observed for those who MPA practiced for at least 10 minutes continuously/week when compared to those who did not do any MPA (Table 3). Those who practiced VPA for at least 10 minutes continuously/week had significantly higher rates of success in the whole mouth salty test (p = 0.018), the whole mouth salty .32 test (p = 0.004) and the whole mouth total score (p = 0.002).

**Table 3:** Moderate and vigorous physical activity practice and their association with the whole mouth test.

		Whole mouth bitter			Whole mouth salty			Whole mouth salty .32			Whole mouth total		
		n	Success %	P value	n	Success %	P value	n	Success %	P value	n	Success %	P value
**Moderate PA**	**Yes**	1343	**84.2**	**0.046**	1342	97.6	0.301	1342	90.2	0.257	1341	**75.5**	**0.018**
	**No**	1771	**81.5**		1770	97,0		1770	89.0		1769	**71.7**	
**Vigourous PA**	**Yes**	511	84.7	0.175	511	**98.8**	**0.018**	510	**93.1**	**0.004**	510	**78.8**	**0.002**
	**No**	2603	82.3		2601	**97.0**		2602	**88.8**		2600	**72.2**	

PA: Physical activity

When assessing MPA’s frequency and duration, one significant difference was noted for frequency of MPA practice per week and for the whole mouth bitter taste test with an OR = 1.059 (p = 0.013) (see Table 4). Significant results for both frequency of practice and duration were observed for VPA: a higher frequency of VPA was associated with significantly better results for bitter (OR = 1.100, p = 0.010), less concentrated salty solution (OR = 1.145, p = 0.009) and total score test (OR = 1.131, p<0.001). Better results for both less concentrated salty solution (OR = 1.021, p = 0.009) and total score (OR = 1.014, p = 0.001) were seen with increased duration of VPA (Table 4).

**Table 4:** Moderate and vigorous physical activity frequency and duration results for the whole mouth test.

		Whole mouth bitter			Whole mouth salty			Whole mouth salty .32			Whole mouth total		
		n	OR [CI]	P value	n	OR [CI]	P value	n	OR [CI]	P value	n	OR [CI]	P value
**Moderate PA**	**Frequency (days/week)**	3114	**1.059 [1.012–1.108]**	**0.013**	3112	1.000 [0.928–1.077]	0.995	3112	1.013 [0.965–1.063]	0.601	3110	1.035 [0.997–1.073]	0.070
	**Duration (10min blocs/week)**	3113	1.006 [0.999–1.012]	0.072	3111	0.995 [0.985–1.005]	0.316	3111	1.003 [0.996–1.010]	0.416	3109	1.003 [0.998–1.008]	0.227
**Vigourous PA**	**Frequency (days/week)**	3114	**1.100 [1.023–1.182]**	**0.010**	3112	1.296 [0.999–1.681]	0.051	3112	**1.145 [1.035–1.266]**	**0.009**	3110	**1.131 [1.062–1.205]**	**<0.001**
	**Duration (10min blocs/week)**	3113	1.009 [1.000–1.019]	0.052	3111	1.047 [0.998–1.099]	0.060	3111	**1.021 [1.005–1.037]**	**0.009**	3109	**1.014 [1.005–1.022]**	**0.001 **

PA: Physical activity

For MPA practice frequency, significantly better results in taste capacity were noted in the obesity 1 BMI category for the bitter test with an OR = 1.136 (p = 0.017) and the whole mouth total score with an OR = 1.130 (p = 0.008). Other results for MPA practice were not significant (data not presented). When looking at the impact of VPA, better taste recognition for the normal and overweight BMI categories were noted with higher PA frequency for the bitter (OR = 1.181, p = 0.017; OR = 1.180, p = 0.019 and total score test (OR = 1.138, p = 0.019; OR = 1.244, p<0.001). VPA Duration was also associated in the normal and overweight BMI categories with better results for bitter (OR = 1.025, p = 0.026; OR = 1.023, p = 0.027) and total score test (OR = 1.017, p = 0.033; OR = 1.032, p<0.001) for the whole mouth test. Also, there was a significant positive association with the less concentrated salty solution, for frequency (OR = 1.305, p = 0.006) in the overweight BMI category (OR = 1.044, p = 0.009). Some negative impacts of VPA were noted for the obesity 2–3 BMI category in the whole mouth bitter test for frequency and duration (OR = 0.802, p = 0.016; OR = 0.957, p = 0.002, respectively), and in the whole mouth total score for duration (OR = 0.964, p = 0.009) (see Table 5).

**Table 5:** Vigorous physical activity frequency and duration results for the whole mouth test stratified for BMI.

		Whole mouth bitter			Whole mouth salty			Whole mouth salty .32			Whole mouth total		
	BMI Categories	n	OR [CI]	P value	n	OR [CI]	P value	n	OR [CI]	P value	n	OR [CI]	P value
**Frequency (days/ week)**	**Normal**	835	1.181 [1.031–1.353]	**0.017**	835	1.870 [0.819–4.266]	0.137	835	1.049 [0.898–1.226]	0.548	832	**1.138 [1.021–1.267]**	**0.019**
	**Overweight**	1044	**1.180 [1.028–1.355]**	**0.019**	1043	1.201 [0.822–1.754]	0.344	1043	**1.305 [1.079–1.578]**	**0.006**	1042	**1.244 [1.106–1.400]**	**<0.001**
	**Obesity 1**	684	1.050 [0.890–1.237]	0.564	684	0.963 [0.654–1.419]	0.849	684	1.524 [0.964–2.409]	0.071	684	1.116 [0.954–1.305]	0.170
	**Obesity 2–3**	523	**0.802 [0.669–0.960]**	**0.016**	522	NA	0.995	523	0.875 [0.696–1.101]	0.254	522	0.848 [0.714–1.009]	0.063
**Duration (10min blocs/ week)**	**Normal**	835	**1.025 [1.003–1.047]**	**0.026**	835	1.122 [0.949–1.325]	0.177	835	1.005 [0.986–1.025]	0.607	835	**1.017 [1.001–1.033]**	**0.033**
	**Overweight**	1043	**1.023 [1.003–1.044]**	**0.027**	1042	1.039 [0.967–1.116]	0.295	1042	**1.044 [1.011–1.079]**	**0.009**	1041	**1.032 [1.013–1.051]**	**<0.001**
	**Obesity 1**	684	0.999 [0.984–1.015]	0.953	684	1.003 [0.958–1.050]	0.895	684	1.118 [0.981–1.274]	0.094	684	1.006 [0.989–1.022]	0.490
	**Obesity 2–3**	523	**0.957 [0.931–0.984]**	**0.002**	522	NA	0.988	523	0.988 [0.955–1.023]	0.504	522	**0.964 [0.939–0.991]**	**0.009**

PA: Physical activity

When analysis was stratified by sex, both frequency and duration of MPA and VPA did not yield significant results for the whole mouth test in the women group (data not shown). In the group comprised of only men, frequency and duration of MPA were associated with better results in the bitter (OR = 1.092, p = 0.012; OR = 1.064, p = 0.029) and total score test (OR = 1.015, p = 0.005; OR = 1.009, p = 0.019) for the whole mouth test. Better results in the bitter (OR = 1.191, p = 0.002), less concentrated salty (OR = 1.191, p = 0.017) and total score salty (OR = 1.204, p<0.001) for the whole mouth test were noted for VPA frequency. As for duration of VPA practice, better results in the bitter (OR = 1.016, p = 0.023), less concentrated salty (OR = 1.033, p = 0.009) and total score test (OR = 1.021, p<0.001) were observed for the whole mouth test.

## 4. Discussion

This study extensively investigated the relationship between overall PA and taste perception and how different components of chronic PA (intensity, frequency, duration) are associated with taste perception. The current findings support a positive association between regular practice of physical activity and the preservation of taste capacities, specifically with activities performed at higher intensity (i.e., VPA). The results reveal that higher frequency or duration of PA were both associated with better taste function results for both the tongue tip taste test and the whole mouth taste test. For the tongue tip taste test, people who practiced at least 10 minutes/week of VPA had better success rates than those who did not. Additionally, frequency and duration of VPA were associated with better results in bitter, salty, and total score tests. For the whole mouth test, better success rates were observed in people who practiced MPA or VPA for at least 10 minutes/week. Furthermore, both MPA and VPA frequency were associated with better results in bitter taste, while both VPA frequency and duration were associated with better results in salty taste and total score tests. Stratified analyses for BMI status showed positive associations mostly between VPA frequency and durations and taste test results for the normal weight and overweight categories. However, some negative associations were specifically noted for the obesity 2–3 BMI category. When looking at the analyses stratified for sex, no significant results were obtained between active and inactive women groups. Positive associations were present for men, especially in those who practiced VPA, for whom better taste results were observed in association with both frequency and duration of PA.

### Frequency

Despite no significant results for the frequency of MPA practice on the tongue tip taste test, the frequency of MPA practice provided better taste results for the bitter whole mouth taste test, with an OR = 1.059. This suggests that for every increment of one day of MPA practice, the probability of successful bitter taste results increases by roughly 6%. In general, VPA practice offered better results in both the tongue tip and the whole mouth taste tests. Regarding VPA’s frequency for the tongue tip taste test, ORs for frequency ranged between 1.055 and 1.061. Similar to MPA, a ~6% increase can be expected for both bitter and total score tests. For the whole mouth taste test, ORs between 1.100 and 1.145 were observed for the frequency of VPA. Therefore, the probability of a successful whole mouth taste test increases by 10% to 15% for each increment of one day of VPA practice per week. In a diabetic population, regular PA was shown to have a positive impact on sweet taste perceptions. In fact, a 6-month intervention which included brisk walking 4–5 days/week (total ≥150minutes) resulted in significant improvement in sweet taste sensitivity while decreasing sweet preference in this population [20]. Chronic physical activity appears to correlate with higher sweet taste intensity ratings compared to inactivity, suggesting that active individuals may have enhanced taste intensity perception [14]. In this study, participants were classified as either inactive (≤1 weekly exercise session) or active (≥4 weekly exercise sessions), with others excluded [14]. Interestingly, no differences in bitterness, saltiness, or sourness ratings between groups were observed [14].

### Duration

While no significant results for the duration of MPA’s practice were observed for the tongue tip taste test, ORs between 1.006 and 1.013 were obtained for the duration of VPA’s practice. This would suggest that for each additional 10 minutes of VPA practice per week, the probability of a successful tongue tip taste test increases by 0.6% up to 1.3%. For the whole mouth taste test, while no significant association was observed for MPA, ORs ranging from 1.014 to 1.021 were observed with the duration of VPA’s practice for the total score test and less concentrated salty score respectively. In this instance, increases in the probability of success would be between 1.4% up to 2.1% for each increment of 10 minutes of VPA practiced per week. This suggest that duration of intensity appears as less important than frequency, at least for the intervals chosen in the current study. The relationship between weekly PA duration and taste perceptions remains unexplored in the literature, although some studies have revealed different outcomes for acute long-duration activities. Notably, Narukawa and colleagues performed two back-to-back studies where a half-marathon or a day-long hike were performed and sweet taste proxies were evaluated [21, 22]. Interestingly, a half-marathon enhanced sweet taste sensitivity (lower detection threshold for sucrose) [21], while no change was detected following a 36-km hike for taste intensity and palatability [22]. These findings might suggest intensity as a potential determinant, with half-marathons typically involving higher intensity than longer hikes. Therefore, future research should consider the interplay of intensity and duration in PA when examining their impacts on taste sensitivity.

### Intensity

Nakanishi and colleagues reported augmented sweet taste sensitivity following acute PA with higher intensity (70% VO_2_MAX), but not with lower intensities (50% VO_2_MAX) [23]. These results put into perspective the possible impact of acute PA’s intensity on taste perception. Martins and colleagues also had an intensity-based PA intervention, in which participant completed isocaloric exercise sessions of moderate-intensity continuous training, high-intensity interval training (HIIT), or short-duration HIIT for 12 weeks [24]. While sweet taste sensitivity or intensity were not tested, the author registered no significant differences between sweet taste preferences between all condition [24]. The current study results report that chronic PA performed at higher intensities might be of great interest in preventing taste dysfunction. While our results on overall PA level require a minimum of 10 minutes per week, it should be noted that, in this sample, the mean PA duration was around 88 minutes/week for MPA and 39 minutes/week for VPA, suggesting that more than 10 minutes might be necessary to obtain positive outcomes for taste. At first, it was questioned whether the sole practice of MPA or VPA, utilizing a dichotomous ’yes-no’ query to document PA practice, could positively be associated to taste test results. For the whole mouth taste test, significantly higher success rates were observed when comparing physically active people with their non-active counterparts for the bitter (2.7% difference) and the total score test (3.8% difference) for MPA practice. Regarding VPA practice, significantly higher success rates for the bitter (6.9% difference), salty (5.2% difference) and the total score test (6.6% difference) for the tongue tip taste test were observed. For the whole mouth taste test, significantly higher success rates were noted for the salty (1.8% difference), less concentrated salty (4.3% difference) and the total score test (6.6% difference). These results confirm that being active for at least 10 minutes of PA per week has benefits concerning taste capacities. Similar results were found by Hoffman and colleagues when assessing the relationship between olfactory function and physical activity in the 2011–2015 NHANES cohort [15]. The authors described that participating in PA, characterized as ’participating in moderate-to-vigorous physical activity ≥3 days in a typical week,’ as having a strong protective effect [15].

### Bodyweight status

When stratified for BMI, while one significant difference was observed for the tongue tip taste test—specifically in VPA practice for normal weight individuals for the bitter taste—multiple results were obtained for specific BMI categories for the whole mouth taste test. Although both frequency and duration of VPA offered better results for the whole mouth taste test, ORs for frequency were higher for the bitter and total score test. In fact, ORs for the frequency of VPA for the overweight BMI category were much higher compared to their duration counterpart (1.180 vs 1.023 and 1.244 vs 1.032, respectively). It is to be noted that the duration was divided into multiple 10-minute blocks, implying that a 2 to 3% increase in the probability of a successful test, in this specific case, is to be expected for every 10 minutes of VPA practice. This would suggest that the frequency of VPA offers greater benefits regarding taste preservation than total duration across the week, but only when each increase is compared in a 1:1 unit increment ratio.

Despite general positive results, both frequency and duration of VPA were also negatively associated with taste function in the obesity 2–3 BMI category for the whole mouth taste test. In contrast, MPA frequency was significantly associated with better results in taste capacity in the obesity 1 BMI category. The results might provide insight into the role of physical activity and its impact on individuals living with obesity, who seemed to benefit from lower intensities in this case. Presumably, normal weight and overweight individuals looking to optimize their sense of taste could prioritize regular VPA, while people living with obesity might benefit more from lower PA intensities. It’s crucial to recognize that achieving such high-intensity levels can pose challenges for those living with excess body weight and mechanisms involved in the adverse impact of VPA might be investigated.

### Sex response

One intriguing result in this paper was that women’s taste did not seem to benefit from an active lifestyle, whether it be from MPA or VPA’s frequency and duration. It’s important to note that sub-analyses revealed that at baseline, significantly higher success rates (3% to 7% higher) were observed for women compared to men in the tongue tip taste test (p<0.001). It has been observed that women usually exhibit better taste perceptions than men across all ages, especially when lifestyle factors are not accounted for [10]. In this case, the initial difference might explain why no significant results were obtained when analyses were stratified by sex. Since women generally exhibit better or preserved taste capacities when compared to men, it could imply that their baseline capacities are de facto higher than most men in this data set. In fact, when examining the differences in success rates in women overall—whether they’re active or not—higher success rates in the tongue tip taste test are observed even when compared to active men. As for the whole mouth taste test, while women overall still exhibit better success rates, active men appear to achieve similar or even superior success rates in some taste tests. In this case, while PA has a positive impact on the men-only group, it could be supposed that it propels them towards what they’re supposed to be exhibiting in the first place. With this logic, the benefits observed from MPA and VPA could only be seen when compared to populations with altered taste perceptions at baseline. While controlled studies and specific protocols are still needed to uncover the role of physical activity on taste perceptions, one could argue that its role is more to maintain proper taste function rather than to elevate it.

### Strengths and limitations

This study is the first to address the relation between chronic PA (overall and specific components) and taste perceptions in over 3000 people, while controlling for different cofactors such as age and sex. Also, this study offered stratified analyses regarding BMI and sex, which both turned out to have different implications when looking at the link between physical activity and taste. While these results offer good guidance on how to optimize sensory preservation, with PA as the main intervention, it is still limited to an association study. This study offers analyses of association between chronic PA and taste, and while age and sex were controlled factors, general lifestyle habits were not accounted for. While these analyses did show a positive relationship between PA frequency and duration, it is unclear if the people who were more active also had overall healthier habits. It is to be noted that most of the benefits of PA’s frequency and duration were observed for normal weight and overweight individuals, mostly regarding vigorous physical activity. Also, previous work done on PA’s impact on taste perceptions mostly included sweet taste or salty taste analyses that were either performed with varying tastant concentrations or for which their intensity was assessed. Although salty taste was present in the current dataset, the taste test that were included in the NHANES protocol have not been observed elsewhere in the taste-PA literature. Examining cerebral responses to different taste concentrations following PA, using EEG data, could provide important insights into how PA affects taste. This would improve our comprehension of PA’s impact on taste perception and open the door for more extensive research in this field.

## 5. Conclusion

The present study provides evidence supporting the association between an active lifestyle, especially frequency and intensity, and taste function. The findings suggest that individuals who engage in regular PA might benefit more from increased frequency and intensity, particularly for men and those with normal bodyweight and overweight status. For individuals living with obesity, the interest of higher intensity is questioned. For women, the absence of association between taste function and PA might be due to a preserved profile compared to men. These results contribute to our understanding on the relationship between PA and sensory function, highlighting the relative importance of specific PA parameters such as intensity, duration and frequency in subgroups of the population. Overall, these findings have implications for promoting an active lifestyle to optimize taste perception and to inform public health interventions.
